# Fat Mass Index Better Identifies Metabolic Syndrome: Insights from Patients in Early Outpatient Cardiac Rehabilitation

**DOI:** 10.3390/jcm8122147

**Published:** 2019-12-05

**Authors:** Amanda R. Bonikowske, Maria Irene Barillas Lara, Katlyn E. Koepp, Jose R. Medina Inojosa, Ray W. Squires, Francisco Lopez-Jimenez, Thomas P. Olson

**Affiliations:** Department of Cardiovascular Medicine, Mayo Clinic, Rochester, MN 55905, USA; mibarillas@ufm.edu (M.I.B.L.); koepp.katlyn@mayo.edu (K.E.K.); medinainojosa.jose@mayo.edu (J.R.M.I.); squires.ray@mayo.edu (R.W.S.); lopez@mayo.edu (F.L.-J.); olson.thomas2@mayo.edu (T.P.O.)

**Keywords:** body mass index, cardiac rehabilitation, fat mass index, metabolic syndrome

## Abstract

Body mass index (BMI) does not differentiate fat and lean mass or the distribution of adipose tissue. The purpose of this study was to examine the prevalence of metabolic syndrome (MetS) among patients entering outpatient cardiac rehabilitation (CR) across fat mass index (FMI) categories compared with BMI. This retrospective cross-sectional study evaluated dual-energy x-ray absorptiometry in 483 CR patients from 1 January 2014, through 31 December 2017. Clinical data were extracted from the electronic health record. Patients were grouped by FMI and BMI categories. Mean (SD) age of patients was 64.3 (14) years. The normal FMI category had 15 patients; excess fat, 74; and obese, 384. In contrast, 93, 174, and 216 were in the normal, overweight, and obese BMI categories, respectively. Prevalence of MetS was 0 (0%) in normal, 5 (1%) in excess fat, and 167 (54%) in obese FMI, with 97% in the obese category. MetS prevalence was 4 patients (0.8%) in normal, 39 (8%) in overweight, and 129 (27%) in obese BMI categories, with 75% of MetS in the obese category. FMI more accurately classified CR patients with metabolically abnormal fat (*p* < 0.001). FMI is a more sensitive index than BMI for metabolically abnormal fat of outpatient CR patients.

## 1. Introduction

Body mass index (BMI) (total body mass as kg/m^2^) has long been used to classify obesity status across the aging and sex spectrums. However, BMI has known limitations for classification of obesity [1]. It neither differentiates between lean mass and fat mass nor provides the distribution of adipose tissue. Obesity is defined as BMI ≥30; however, there are no universally accepted normative ranges for body fat percentage. Sex- and age-specific categories have been reported, as well as ranges for optimal health of 10% to 22% and 20% to 32% for men and women, respectively [2]. Other investigators have defined thresholds of ≥25% body fat for men and ≥32% for women [3,4]. Additionally, central adipose tissue deposition is associated with greater metabolic abnormalities, specifically metabolic syndrome (MetS) and increased cardiovascular risk, than gluteal-femoral fat distribution [5]. Moreover, adipose tissue distribution is known to differ between the sexes: men have more central distribution compared with the gluteal-femoral distribution of women [6].

The cluster of risk factors associated with MetS, in combination, increases the risk of multiple chronic diseases, including cardiovascular disease [7,8,9]. The diagnosis of MetS is dependent on adiposity distribution, defined by increased waist circumference and metabolically relevant risk factors such as elevated triglyceride level, decreased high-density lipoprotein cholesterol (HDL-C), increased blood pressure, and increased fasting blood glucose [10,11]. An estimated one-third of US adults and approximately one-half of patients entering cardiac rehabilitation (CR) have MetS [12,13].

An apparent obesity paradox has been reported, suggesting that obesity, defined with BMI, is protective of death for patients with cardiovascular disease [14]. In contrast, recent data indicate a lack of paradox when body adiposity is measured objectively [15]. Therefore, in the use of more accurate measures of body composition, such as dual-energy x-ray absorptiometry (DXA), the prevalence of MetS and the relationship between fat mass and lean mass can be determined accurately. Subsequently, the fat mass index (FMI) (total fat mass as kg/m^2^) was developed with the use of DXA data from a large cohort of the National Health and Nutrition Examination Survey [16]. FMI classification thresholds are prevalence matched to BMI classifications, but they identify groups on the basis of fat mass as severe, moderate, or mildly deficit; normal; excess fat; and obese classes I, II, and III [16].

Compared with BMI, FMI is a sex-specific novel metric for the evaluation of body composition that offers greater precision in the determination of fat mass quantity among persons with metabolic abnormalities. FMI may provide greater accuracy in the description of relationships between MetS and so-called true obesity among patient groups. This more accurate assessment of obesity and its relationship with MetS may be useful particularly for risk stratification of patients entering outpatient CR, to more accurately develop individualized treatment plans, exercise prescriptions, and nutritional interventions.

The purpose of the present study was to compare two measures of adiposity, FMI and BMI, with the prevalence of MetS and its components among patients entering outpatient CR. We hypothesized that the distribution of MetS and its components is more pronounced at higher FMI categories and with a greater proportion of patients who have MetS at lower BMI categories.

## 2. Materials and Methods

### 2.1. Study Design and Population

This retrospective cross-sectional study evaluated patients entering CR from among the 1121 who entered and completed CR during the study period. The study patients completed a baseline DXA between January 2014 and December 2017. Clinical data were collected from the Mayo Clinic CR database and electronic health records and included sex, height, weight, waist circumference, blood pressure, fasting blood glucose, triglycerides, HDL-C, cardiovascular medications, and cardiovascular diagnoses. The Mayo Clinic Institutional Review Board approved this study, and as required by Minnesota law, all study patients provided authorization for use of their health records.

### 2.2. Anthropometric Measurements

Trained radiology technicians conducted DXA (Lunar iDXA Series X; GE Healthcare) using standardized procedures recommended by the manufacturer, as previously described [17]. All DXA scans were performed within 2 weeks of the initiation of CR program activities. The scans were used to measure total body mass, total body fat mass, total body lean mass, regional distribution of fat and lean masses, and percentage of fat mass for total and regional distributions.

The abdominal region was defined as the area between the ribs and pelvis, as recommended by the manufacturer’s software application. Patients were classified on the basis of standard FMI classification thresholds [16]. FMI categories for male patients were <3 (mild, moderate, and severe fat deficit), 3 to 6 (normal), >6 to 9 (excess fat (overweight)), and >9 (obesity classes I, II, and III) [16]. Categories for women were FMI <5 (mild, moderate, and severe fat deficit), 5 to 9 (normal), >9 to 13 (excess fat), and >13 (obesity classes I, II, and III) [16]. By comparison, for BMI, patients were classified on the basis of reference BMI classification thresholds [18]. BMI categories were underweight, BMI <18.5; normal weight, 18.5 to 24.9; overweight, 25.0 to 29.9, and obese, ≥30.0 [19].

### 2.3. Metabolic Syndrome

The Adult Treatment Panel III guidelines defined MetS [20]. These guidelines cite five criteria, with diagnosis based on the presence of three or more criteria: waist circumference >35 inches for women and >40 inches for men, fasting triglycerides ≥150 mg/dL, fasting HDL-C <40 mg/dL in men and <50 mg/dL in women, blood pressure ≥130/85 mm Hg, and fasting blood glucose ≥110 mg/dL [20,21].

### 2.4. Statistical Methods

All analyses were completed with JMP version 13.0 (SAS Institute Inc, Cary, NC, USA). Continuous variables were reported as mean (SD) and categorical variables as frequency and percentage. Comparison of continuous variables was conducted with analysis of variance (ANOVA) or *t*-test; categorical data were compared between groups with Pearson χ^2^ or Fisher exact test. Due to the expected high prevalence of MetS, a log-binomial regression model was used to evaluate the age and sex adjusted odds ratios of having MetS across FMI and BMI categories. Statistical significance was set at the two-tailed α level of 0.05.

## 3. Results

### 3.1. Patient Population

The patients undergoing CR had a mean (SD) age of 64.3 (14) years. Most patients were residents of Olmsted County, Minnesota, and 97% were non-Hispanic Caucasian (Table 1 and Table 2).

### 3.2. Anthropometrics

**Figure 1:**
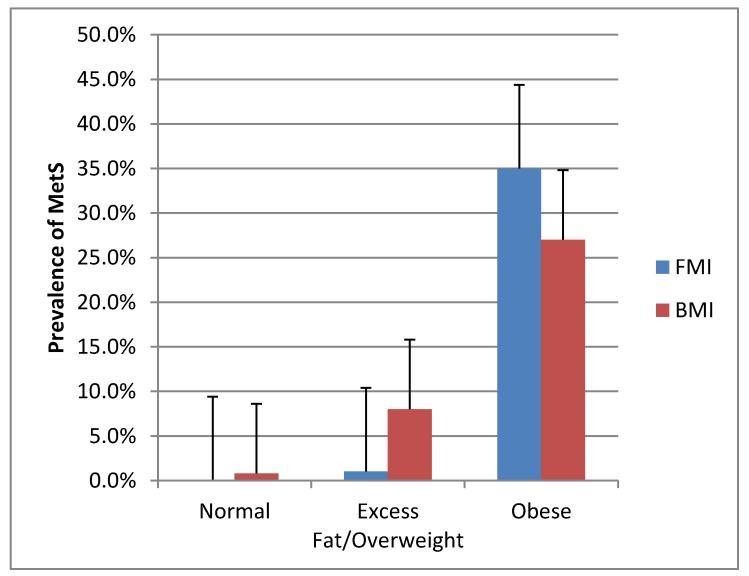
Prevalence of MetS for the fat mass index (FMI) and body mass index (BMI) categories.

The FMI categories of normal, excess fat, and obese had 15, 74, and 394 patients, respectively. By comparison, the BMI categories of normal, overweight, and obese had 93, 174, and 216 patients. The prevalence of MetS with use of FMI was 0 patients (0%) in normal, 5 (1%) in excess fat, and 169 (35%) in obese groups (Figure 1). With use of BMI, the MetS prevalence was 4 patients (0.8%) in normal, 39 (8.1%) in overweight, and 131 (27.1%) in obese groups. The MetS proportion was greatest in the obese category for men and women when either method was used (*p* < 0.05). Men and women with MetS had more fat mass and less fat-free mass than those without MetS (*p* < 0.05).

### 3.3. Metabolic Syndrome

**Figure 2:**
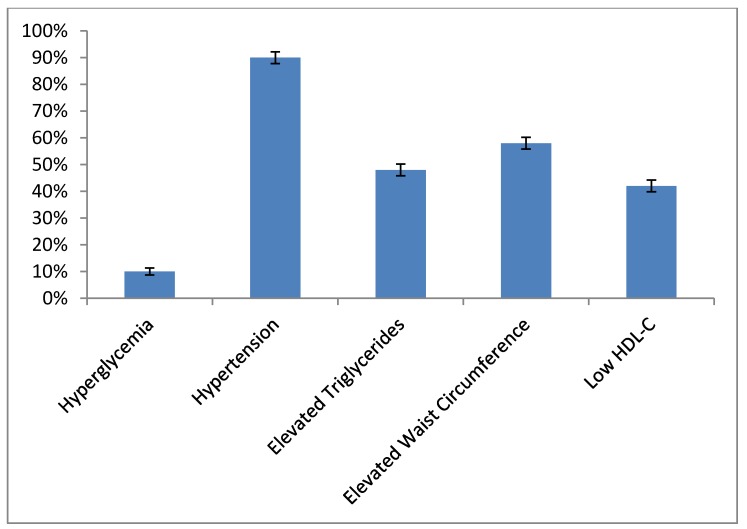
Prevalence of metabolic syndrome (MetS) components. HDL-C, high-density lipoprotein cholesterol.

**Figure 3:**
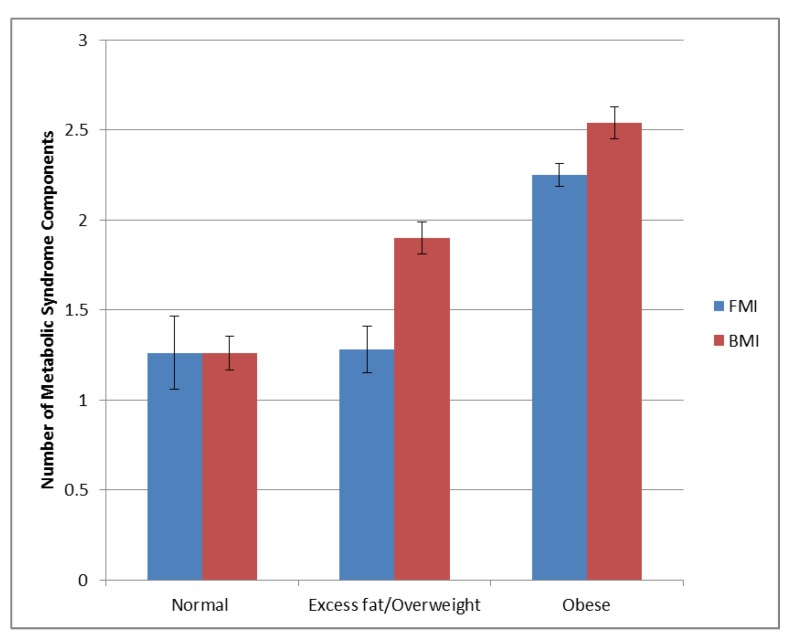
Mean number of MetS components for FMI and BMI categories. Error bars represent standard error.

The overall prevalence of MetS was 35%, with no difference in prevalence between women (31.5%) and men (36.4%) (*p* = 0.30). Figure 2 shows the prevalence of MetS components. Results of the 2 × 3 ANOVA show the superiority of FMI vs. BMI in the classification of patients into the metabolically abnormal categories (*p* < 0.001). Figure 1 shows the absence of MetS in the normal FMI category. With the use of FMI, 97% of MetS was in the obese category vs. 75% with the use of BMI. Furthermore, when compared to overweight individuals, the likelihood of having MetS was higher in those obese by FMI (age and sex adjusted odds ratio (OR) = 3.26; 95% confidence interval (CI) 2.95–3.62, *p =* 0.0002) compared to those obese by BMI (age and sex adjusted OR = 2.84; 95% CI 1.23–6.56, *p* < 0.0001). Figure 3 shows the average number of MetS components in the FMI and BMI categories.

## 4. Discussion

The novel finding of the present study is that the use of FMI calculated from DXA measurements correctly classified CR patients with and without MetS into the appropriate fat mass categories. The classification of men and women in the obese category nearly doubled when FMI was used since the use of fat mass relative to height is not confounded by lean mass, which is the case with BMI. This study showed a greater frequency of metabolic abnormalities with body fat content greater than normal or excess fat.

The FMI was developed in response to a need for a measure not confounded by lean mass. It more accurately describes the extent of fat accumulation. Previous work has shown that FMI is positively and independently associated with the presence of MetS among apparently healthy adults, and this observation is independent of BMI and body fat percentage [22]. Conversely, the lean mass index may be useful in the differentiation of persons at higher risk of MetS and may help to provide individualized lifestyle management recommendations.

In the present study, we observed that patients with MetS and a higher FMI also had less lean mass. The FMI aids in providing an individualized-medicine approach to patient care because some patients with normal BMI may have MetS. Body composition abnormalities are associated with different disease presentations. Visceral adipose tissue and muscle fat infiltrate are associated with coronary artery disease (CAD) and type 2 diabetes mellitus, whereas high liver fat content is associated with type 2 diabetes mellitus solely. Conversely, low liver fat content has been associated with CAD [23].

The association of fat distribution to metabolic status is complex and cannot be explained with simple single-compartment models such as BMI. Individual assessment based on specific fat distribution allows for more rapid identification of increased risk and thereby allows for early targeted and effective treatment strategies tailored to individual patient needs.

According to the obesity paradox, a higher BMI may be protective against CAD. However, recent findings suggest that this paradox may be a fallacy [15]. Medina-Inojosa et al. [15] suggest that the number of major adverse cardiovascular events is greater with increased body fat percentage and decreased lean body mass—not simply with increased BMI—which shows the importance of assessment of fat and lean mass. Our results are consistent with their data in that our cohort had greater metabolic abnormalities in the excess fat and obese groups when FMI was used for analysis than with BMI use [23].

The FMI facilitates accurate classification of patients into fat mass categories and may more precisely identify those at increased risk for future heart failure, atrial fibrillation, and death [24,25,26,27,28]. Quantification of fat and lean mass provides additional prognostic information because lean mass is associated with lower risk of major adverse cardiovascular events [15]. In CR programs, clinical staff can apply this knowledge in the design of effective individualized treatment plans, exercise prescriptions, and nutritional interventions aimed at increasing lean mass and reducing fat mass. Approaches can be individualized for patients with obesity compared with those with obesity and concurrent MetS.

Data from other investigators show that a portion of the CR population is obese yet does not have MetS (i.e., approximately 30–35% may be so-called metabolically healthy obese (MHO)) [29]. Our data corroborate previous findings; approximately 47% of our obese patients did not have overt MetS. Yet, MHO adults without known CADare at increased risk for CAD events compared with metabolically healthy normal-weight adults but at a lower risk than metabolically unhealthy obese and metabolically unhealthy normal-weight adults [30,31]. In addition, MHO persons are at greater risk of metabolic abnormalities than their metabolically healthy normal-weight counterparts [29]. Of note, the definition of MHO is not standardized, and a benign obesity phenotype has not been identified [31].

Interestingly, the overall prevalence of MetS (35%) in this CR population is lower than previously reported for other cohorts. MetS prevalence was 50% and 58% in two separate CR program populations [12,32] and was 51% in a cohort of patients referred for elective coronary angiography [33]. The lower prevalence may represent a leveling off of increasing obesity rates and MetS prevalence after aggressive preventive measures since approximately 2003 [34]. Demographic, geographic, and socioeconomic factors may explain the lower prevalence of MetS in our CR population. Olmsted County, Minnesota has a high median household income ($72,337) and low poverty (8.3%) with access to numerous healthcare facilities (US Census Bureau, 2013–2017). However, our population had a higher hypertension prevalence of approximately 90% than the 60% to 70% prevalence in prior studies [12]. The importance of addressing and treating MetS in the CR population is related directly to the increased risk of heart failure, because hypertension has been identified as a central factor in future heart failure for persons with combined CAD and MetS [35].

### Limitations

A limitation of the study is the non-Hispanic Caucasian homogeneity of the patient population, which is a known potential limitation of all retrospective observational studies. Extrapolation of current results to more diverse patient populations is not possible. Selection bias may be present because of the inclusion of patients who were willing to have DXA at the start of outpatient CR.

## 5. Conclusions

FMI was more accurate than BMI for the classification of patients into appropriate body composition categories. It also was more sensitive in the detection of patients with MetS. The proportion of the population in the overweight and obese categories was greater with the use of FMI than BMI. More patients with MetS were identified in the overweight and obese FMI categories than in the BMI categories, with the greatest MetS prevalence in the obese FMI category. Although MetS can be detected even when BMI is normal, no MetS was present among patients classified as normal with FMI in the present study. Therefore, FMI is a more accurate index of metabolically abnormal body fat.

## Figures and Tables

**Table 1:** Patient characteristics by body mass index categories.

	Normal	Overweight	Obese	All	*p-*Value
*n* (%)	93 (19%)	174 (36%)	216 (45%)	483 (100%)	
Age, years	63 ± 16	66 ± 13	63 ± 13	64 ± 14	0.0416 *
Height, cm	169 ± 9	171 ± 9	172 ± 9	171 ± 9	0.1457
Weight, kg	64 ± 9	80 ± 11	103 ± 16	87 ± 20	<0.0001 *
Body mass index	22 ± 2	27 ± 2	35 ± 4	30 ± 6	<0.0001 *
Female	34 (37%)	44 (25%)	64 (30%)	142 (29%)	0.1597
Waist, cm	86 ± 8	98 ± 8	116 ± 11	104 ± 15	<0.0001 *
Hip, cm	95 ± 5	103 ± 5	116 ± 10	107 ± 12	<0.0001 *
Resting Blood pressure					
Systolic, mmHg	120 ± 22	118 ± 18	120 ± 16	120 ± 19	0.2164
Diastolic, mmHg	67 ± 12	68 ± 12	69 ± 12	68 ± 12	0.0462 *
DXA Variables					
Total body mass, kg	65 ± 9	80 ± 10	103 ± 16	87 ± 20	<0.0001 *
Total fat mass, kg	20 ± 14	26 ± 6	42 ± 10	32 ± 13	<0.0001 *
Total lean mass, kg	45 ± 7	50 ± 8	57 ± 10	53 ± 10	<0.0001 *
Body fat, %	27 ± 7	34 ± 6	42 ± 7	36 ± 8	<0.0001 *
Trunk					
Total mass, kg	32 ± 5	41 ± 6	54 ± 9	45 ± 12	<0.0001 *
Fat mass, kg	9 ± 3	15 ± 3	26 ± 7	19 ± 8	<0.0001 *
Lean mass, kg	22 ± 3	24 ± 4	27 ± 5	25 ± 5	<0.0001 *
Fat %	29 ± 9	39 ± 6	49 ± 6	41 ± 10	<0.0001 *
Android					
Total mass, kg	5 ± 1	6 ± 1	9 ± 2	7 ± 2	<0.0001 *
Fat mass, kg	1.4 ± 0.7	2.7 ± 0.7	4.7 ± 1.3	3.3 ± 1.7	<0.0001 *
Lean mass, kg	3.3 ± 0.6	3.7 ± 0.7	4.3 ± 0.9	3.9 ± 0.9	<0.0001 *
Fat %	29 ± 11	41 ± 7	52 ± 7	44 ± 11	<0.0001 *
Gynoid					
Total mass, kg	9 ± 1	11 ± 2	15 ± 3	13 ± 3	<0.0001 *
Fat mass, kg	3 ± 0.9	4 ± 1	7 ± 7	5 ± 5	<0.0001 *
Lean mass, kg	6 ± 1	7 ± 1	8 ± 1	8± 2	<0.0001 *
Fat %	30 ± 8	35 ± 7	42 ± 8	37 ± 9	<0.0001 *
Other Characteristics					
Metabolic syndrome	4 (4%)	39 (22%)	129 (60%)	172 (36%)	<0.0001 *
Current smoker	5 (5%)	13 (7%)	11 (5%)	29 (6%)	0.4653
Hyperglycemia	6 (6%)	11 (6%)	32 (15%)	49 (10%)	0.0093 *
Dyslipidemia	20 (21%)	87 (50%)	126 (58%)	233 (48%)	<0.0001 *
Hypertension	87 (93%)	159 (91%)	190 (88%)	436 (90%)	0.2521
Low HDL	17 (18%)	75 (43%)	113 (52%)	205 (42%)	<0.0001 *
Hypertriglyceridemia	20 (21%)	87 (50%)	126 (58%)	233 (48%)	<0.0001 *
Elevated waist	5 (5%)	75 (43%)	200 (93%)	280 (58%)	<0.0001 *
Medications					
ASA	77 (83%)	154 (88%)	188 (87%)	419 (87%)	0.4331
Plavix	36 (39%)	80 (46%)	112 (52%)	228 (47%)	0.0955
Beta blocker	70 (75%)	145 (83%)	191 (88%)	406 (84%)	0.0170 *
ACE/ARB	36 (39%)	78 (45%)	121 (56%)	235 (49%)	0.0089 *
Statin	66 (71%)	147 (85%)	185 (86%)	398 (82%)	0.0086 *
CR Reasons					
Angina/CAD	3 (3%)	10 (6%)	13 (6%)	26 (5%)	0.0569
CABG	11 (12%)	24 (14%)	34 (16%)	69 (14%)	0.3565
CHF	4 (4%)	9 (5%)	11 (5%)	24 (5%)	0.3565
NSTEMI	13 (14%)	29 (17%)	30 (14%)	72 (15%)	0.3565
Other	6 (6%)	8 (5%)	9 (4%)	23 (5%)	0.3565
PAD	0 (0%)	0 (0%)	1 (0.5%)	1 (0.2%)	0.3565
PCI	16 (17%)	36 (21%)	62 (29%)	114 (24%)	0.3565
STEMI	15 (16%)	26 (15%)	25 (12%)	66 (14%)	0.3565
Ventricular assist device	8 (9%)	9 (5%)	4 (2%)	21 (4%)	0.3565
Valve disease	17 (18%)	23 (13%)	27 (12%)	67 (14%)	0.3565

Data presented as mean ± SD or *n* (%). Abbreviations: BMI, body mass index (kg/m^2^); DXA, dual-energy x-ray absorptiometry; HDL, high-density lipoprotein; ASA, aspirin; ACE, angiotensin-converting enzyme; ARB, angiotensin II receptor blocker; CR, cardiac rehabilitation; CAD, coronary artery disease; CABG, coronary artery bypass grafting; CHF, chronic heart failure; NSTEMI, non-ST-elevation myocardial infarction; PAD, peripheral artery disease; PCI, percutaneous coronary intervention; STEMI, ST-elevation myocardial infarction. Elevated waist = female >89 cm (35 inches) and male >102 cm (40 inches); hyperglycemia ≥150 mg/dL; * denotes statistical significance *p* < 0.05.

**Table 2:** Patient characteristics by fat index categories.

	Normal	Excess Fat	Obese	All	*p-*Value
*n* (%)	15 (3%)	74 (15%)	394 (81%)	483 (100%)	
Age, years	53 ± 19	60 ± 14	62 ± 12	61 ± 13	0.1014
Height, cm	170 ± 8	172 ± 10	171 ± 9	171 ± 9	0.6024
Weight, kg	60 ± 11	73 ± 14	91 ± 20	87 ± 20	<0.0001 *
Body mass index	21 ± 3	25 ± 4	31 ± 5	30 ± 6	<0.0001 *
Female	6 (40%)	23 (31%)	113 (29%)	142 (29%)	0.6173
Waist, cm	80 ± 9	90 ± 10	107 ± 14	104 ± 15	<0.0001 *
Hip, cm	92 ± 7	99 ± 7	110 ± 11	107 ± 12	<0.0001 *
Resting Blood pressure					
Systolic, mmHg	117 ± 15	117 ± 17	123 ± 20	122 ± 20	0.1397
Diastolic, mmHg	72 ± 11	70 ± 9	71 ± 14	71 ± 14	0.8213
DXA Variables					
Total body mass, kg	60 ± 11	73 ± 14	91 ± 19	87 ± 20	<0.0001 *
Total fat mass, kg	21 ± 29	21 ± 10	35 ± 12	32 ± 13	<0.0001 *
Total lean mass, kg	48 ± 9	51 ± 10	53 ± 10	53 ± 10	0.0474 *
Body fat, %	17 ± 4	28 ± 6	39 ± 7	37 ± 9	<0.0001 *
Trunk					
Total mass, kg	29 ± 5	37 ± 8	48 ± 11	45 ± 12	<0.0001 *
Fat mass, kg	4 ± 2	11 ± 5	21 ± 7	19 ± 8	<0.0001 *
Lean mass, kg	23 ± 4	25 ± 4	25 ± 5	25 ± 5	0.3245
Fat %	15 ± 4	30 ± 8	45 ± 7	41 ± 10	<0.0001 *
Android					
Total mass, kg	4 ± 1	6 ± 1	8 ± 2	7 ± 2	<0.0001 *
Fat mass, kg	0.5 ± 0.3	2 ± 0.8	4 ± 1	3 ± 2	<0.0001 *
Lean mass, kg	4 ± 0.8	4 ± 0.8	4 ± 0.9	4 ± 0.9	0.1473
Fat %	13 ± 4	30 ± 9	47 ± 8	44 ± 11	<0.0001 *
Gynoid					
Total mass, kg	9 ± 2	11 ± 2	13 ± 3	13 ± 3	<0.0001 *
Fat mass, kg	2 ± 0.4	3 ± 1	5 ± 5	4 ± 4	<0.0001 *
Lean mass, kg	6 ± 1	7 ± 1	8 ± 2	8 ± 1	0.0019 *
Fat %	21 ± 6	31 ± 8	39 ± 8	37 ± 9	<0.0001 *
Other Characteristics					
Metabolic syndrome	0 (0%)	5 (7%)	167 (42%)	172 (46%)	<0.0001 *
Current smoker	0 (0%)	3 (4%)	26 (7%)	29 (6%)	0.5039
Hyperglycemia	0 (0%)	3 (4%)	46 (12%)	49 (10%)	0.0184 *
Dyslipidemia	4 (27%)	18 (24%)	211 (54%)	233 (48%)	<0.0001 *
Hypertension	13 (87%)	62 (84%)	361 (92%)	436 (90%)	0.1304
Low HDL	3 (20%)	18 (24%)	184 (47%)	205 (42%)	0.0002 *
Hypertriglyceridemia	4 (27%)	18 (24%)	211 (54%)	233 (48%)	<0.0001 *
Elevated waist	2 (13%)	12 (16%)	266 (68%)	280 (58%)	<0.0001 *
Medications					
ASA	11 (73%)	63 (85%)	345 (88%)	419 (87%)	0.3179
Plavix	6 (40%)	30 (41%)	192 (49%)	228 (47%)	0.3656
Beta blocker	14 (93%)	52 (70%)	340 (86%)	406 (84%)	0.0032 *
ACE/ARB	4 (27%)	25 (34%)	206 (52%)	235 (49%)	0.0037 *
Statin	9 (60%)	54 (73%)	335 (85%)	398 (82%)	0.0063 *
CR Reasons					
Angina/CAD	1 (7%)	4 (5%)	21 (5%)	26 (5%)	0.232
CABG	2 (13%)	7 (9%)	60 (15%)	69 (14%)	0.5291
CHF	2 (13%)	3 (4%)	19 (5%)	24 (5%)	0.9731
NSTEMI	1 (7%)	16 (22%)	55 (14%)	72 (15%)	0.9731
Other	1 (7%)	4 (5%)	18 (5%)	23 (5%)	0.9731
PAD	0 (0%)	0 (0%)	1 (0.25%)	1 (0.21%)	0.9731
PCI	3 (20%)	10 (14%)	101 (26%)	114 (24%)	0.9731
STEMI	1 (7%)	14 (19%)	51 (13%)	66 (14%)	0.9731
Ventricular sssist device	2 (13%)	5 (7%)	14 (4%)	21 (4%)	0.9731
Valve disease	2 (13%)	11 (15%)	54 (14%)	67 (14%)	0.9731

Data presented as mean ± SD or *n* (%). CR, cardiac rehabilitation; BMI, body mass index (kg/m^2^). Elevated Waist = female >89 cm (35 inches) and male >102 cm (40 inches); hyperglycemia ≥150 mg/dL; * denotes statistical significance *p* < 0.05.

## Abbreviations

ANOVA	analysis of variance
BMI	body mass index
CAD	coronary artery disease
CR	cardiac rehabilitation
DXA	dual-energy x-ray absorptiometry
FMI	fat mass index
HDL-C	high-density lipoprotein cholesterol
MetS	metabolic syndrome
MHO	metabolically healthy obese

## References

[1] Romero-Corral A., Somers V.K., Sierra-Johnson J., Thomas R.J., Collazo-Clavell M.L., Korinek J.E., Allison T.G., Batsis J.A., Sert-Kuniyoshi F.H., Lopez-Jimenez F. (2008). Accuracy of body mass index in diagnosing obesity in the adult general population. Int. J. Obes. Lond..

[2] Lohman T.G. (1982). Body Composition Methodology in Sports Medicine. Phys. Sportsmed..

[3] Okorodudu D.O., Jumean M.F., Montori V.M., Romero-Corral A., Somers V.K., Erwin P.J., Lopez-Jimenez F. (2010). Diagnostic performance of body mass index to identify obesity as defined by body adiposity: A systematic review and meta-analysis. Int. J. Obes. Lond..

[4] Romero-Corral A., Somers V.K., Sierra-Johnson J., Korenfeld Y., Boarin S., Korinek J., Jensen M.D., Parati G., Lopez-Jimenez F. (2010). Normal weight obesity: A risk factor for cardiometabolic dysregulation and cardiovascular mortality. Eur. Heart J..

[5] Mottillo S., Filion K.B., Genest J., Joseph L., Pilote L., Poirier P., Rinfret S., Schiffrin E.L., Eisenberg M.J. (2010). The metabolic syndrome and cardiovascular risk a systematic review and meta-analysis. J. Am. Coll. Cardiol..

[6] Karastergiou K., Smith S.R., Greenberg A.S., Fried S.K. (2012). Sex differences in human adipose tissues—The biology of pear shape. Biol. Sex. Differ..

[7] Bjørge T., Lukanova A., Jonsson H., Tretli S., Ulmer H., Manjer J., Stocks T., Selmer R., Nagel G., Almquist M. (2010). Metabolic syndrome and breast cancer in the me-can (metabolic syndrome and cancer) project. Cancer Epidemiol. Biomark. Prev..

[8] Ford E.S. (2005). Risks for all-cause mortality, cardiovascular disease, and diabetes associated with the metabolic syndrome: A summary of the evidence. Diabetes Care.

[9] Wu S.H., Liu Z., Ho S.C. (2010). Metabolic syndrome and all-cause mortality: A meta-analysis of prospective cohort studies. Eur. J. Epidemiol..

[10] Grundy S.M., Brewer H.B., Cleeman J.I., Smith S.C., Lenfant C. (2004). Definition of metabolic syndrome: Report of the National Heart, Lung, and Blood Institute/American Heart Association conference on scientific issues related to definition. Circulation.

[11] Expert Panel on Detection E and Treatment of High Blood Cholesterol in A (2001). Executive Summary of The Third Report of The National Cholesterol Education Program (NCEP) Expert Panel on Detection, Evaluation, And Treatment of High Blood Cholesterol In Adults (Adult Treatment Panel III). JAMA.

[12] Savage P.D., Banzer J.A., Balady G.J., Ades P.A. (2005). Prevalence of metabolic syndrome in cardiac rehabilitation/secondary prevention programs. Am. Heart J..

[13] Moore J.X., Chaudhary N., Akinyemiju T. (2017). Metabolic Syndrome Prevalence by Race/Ethnicity and Sex in the United States, National Health and Nutrition Examination Survey, 1988–2012. Prev. Chronic. Dis..

[14] Romero-Corral A., Montori V.M., Somers V.K., Korinek J., Thomas R.J., Allison T.G., Mookadam F., Lopez-Jimenez F. (2006). Association of bodyweight with total mortality and with cardiovascular events in coronary artery disease: A systematic review of cohort studies. Lancet.

[15] Medina-Inojosa J.R., Somers V.K., Thomas R.J., Jean N., Jenkins S.M., Gomez-Ibarra M.A., Supervia M., Lopez-Jimenez F. (2018). Association between Adiposity and Lean Mass with Long-Term Cardiovascular Events in Patients with Coronary Artery Disease: No Paradox. J. Am. Heart Assoc..

[16] Kelly T.L., Wilson K.E., Heymsfield S.B. (2009). Dual energy X-Ray absorptiometry body composition reference values from NHANES. PLoS ONE.

[17] GE Healthcare (2009). Lunar enCORE Safety and Specification Manual.

[18] Garrow J.S., Webster J. (1985). Quetelet's index (W/H2) as a measure of fatness. Int. J. Obes..

[19] National Heart, Lung, Blood Institute, National Institute of Diabetes, Digestive, Kidney Diseases (US) (1998). Clinical Guidelines on the Identification, Evaluation, and Treatment of Overweight and Obesity in Adults: The Evidence Report.

[20] Beilby J. (2004). Definition of Metabolic Syndrome: Report of the National Heart, Lung, and Blood Institute/American Heart Association Conference on Scientific Issues Related to Definition. Clin. Biochem. Rev..

[21] Huang P.L. (2009). A comprehensive definition for metabolic syndrome. Dis. Model. Mech..

[22] Liu P., Ma F., Lou H., Liu Y. (2013). The utility of fat mass index vs. body mass index and percentage of body fat in the screening of metabolic syndrome. BMC Public Health.

[23] Linge J., Borga M., West J., Tuthill T., Miller M.R., Dumitriu A., Thomas E.L., Romu T., Tunón P., Bell J.D. (2018). Body Composition Profiling in the UK Biobank Imaging Study. Obesity.

[24] Zheng R., Zhou D., Zhu Y. (2016). The long-term prognosis of cardiovascular disease and all-cause mortality for metabolically healthy obesity: A systematic review and meta-analysis. J. Epidemiol. Community Health.

[25] Lee H., Choi E.K., Lee S.H., Han K.D., Rhee T.M., Park C.S., Lee S.R., Choe W.S., Lim W.H., Kang S.H. (2017). Atrial fibrillation risk in metabolically healthy obesity: A nationwide population-based study. Int. J. Cardiol..

[26] Lavie C.J., De Schutter A., Patel D.A., Romero-Corral A., Artham S.M., Milani R.V. (2012). Body composition and survival in stable coronary heart disease: Impact of lean mass index and body fat in the “obesity paradox”. J. Am. Coll. Cardiol..

[27] Coutinho T., Goel K., De Sá D.C., Carter R.E., Hodge D.O., Kragelund C., Kanaya A.M., Zeller M., Park J.S., Kober L. (2013). Combining body mass index with measures of central obesity in the assessment of mortality in subjects with coronary disease: Role of “normal weight central obesity”. J. Am. Coll. Cardiol..

[28] Heitmann B.L., Erikson H., Ellsinger B.M., Mikkelsen K.L., Larsson B. (2000). Mortality associated with body fat, fat-free mass and body mass index among 60-year-old swedish men-a 22-year follow-up. The study of men born in 1913. Int. J. Obes. Relat. Metab. Disord..

[29] Lin H., Zhang L., Zheng R., Zheng Y. (2017). The prevalence, metabolic risk and effects of lifestyle intervention for metabolically healthy obesity: A systematic review and meta-analysis: A PRISMA-compliant article. Med. Baltim..

[30] Caleyachetty R., Thomas G.N., Toulis K.A., Mohammed N., Gokhale K.M., Balachandran K., Nirantharakumar K. (2017). Metabolically Healthy Obese and Incident Cardiovascular Disease Events Among 3.5 Million Men and Women. J. Am. Coll. Cardiol..

[31] Eckel N., Meidtner K., Kalle-Uhlmann T., Stefan N., Schulze M.B. (2016). Metabolically healthy obesity and cardiovascular events: A systematic review and meta-analysis. Eur. J. Prev. Cardiol..

[32] Milani R.V., Lavie C.J. (2003). Prevalence and profile of metabolic syndrome in patients following acute coronary events and effects of therapeutic lifestyle change with cardiac rehabilitation. Am. J. Cardiol..

[33] Solymoss B.C., Bourassa M.G., Lespérance J., Levesque S., Marcil M., Varga S., Campeau L. (2003). Incidence and clinical characteristics of the metabolic syndrome in patients with coronary artery disease. Coron. Artery Dis..

[34] Hales C.M., Carroll M.D., Fryar C.D., Ogden C.L. (2017). Prevalence of Obesity Among Adults and Youth: United States, 2015–2016. NCHS Data Brief.

[35] Wang J., Sarnola K., Ruotsalainen S., Moilanen L., Lepistö P., Laakso M., Kuusisto J. (2010). The metabolic syndrome predicts incident congestive heart failure: A 20-year follow-up study of elderly Finns. Atherosclerosis.

